# Impacts of Chopped Food on Primate Behavior Are Not Clear Cut. A Case Study on Zoo‐Housed Ring‐Tailed Lemurs

**DOI:** 10.1002/zoo.70001

**Published:** 2025-06-30

**Authors:** Adam J. George, Samuel Tull, Paul Rose

**Affiliations:** ^1^ School of Sciences Bath Spa University Bath UK; ^2^ University Centre Somerset, Bridgwater & Taunton College Somerset UK; ^3^ Centre for Research in Animal Behaviour, Psychology University of Exeter Devon UK; ^4^ WWT, Slimbridge Wetland Centre, Slimbridge Gloucestershire UK

**Keywords:** activity budgets, behavioral diversity, food presentation, foraging behavior, ring‐tailed lemurs

## Abstract

Measuring how food presentation influences behavior helps encourage practices that support natural foraging efforts for species housed in zoos. To test the effect of food presentation on a commonly housed zoo primate, observations of the behavior of ring‐tailed lemurs (*Lemur catta*), housed at Tropiquaria Zoo in the UK, were undertaken to analyze how different food presentation conditions elicited variation in activity budgets, positive behavioral diversity, and performance of foraging behavior. Lemur behaviors were analyzed in relation to two food presentation conditions (chopped produce and whole produce) using a Shannon's Diversity Index (*H*‐index) adapted for behavioral data. A repeated measures ANOVA was used to investigate whether factors including weather, temperature, and visitor presence had an impact on foraging behavior or if food presentation style was the most important factor. Higher rates of foraging and exploration (locomotion) were observed when lemurs were fed chopped food, whereas animals spent more time eating and performing maintenance behaviors when provided with whole food. There was no significant difference in calculated positive behavioral diversity between chopped and whole food. Food presentation style and weather conditions were important influences of time spent foraging, but temperature and visitor presence had no effect. Our findings show how other influencing factors, alongside of food presentation style, are likely to affect how zoo animals engage with, and ultimately consume, the diet they are offered. We suggest that providing zoo‐housed lemurs with both chopped and whole food items is likely to promote a range of natural foraging behaviors and enhance overall animal welfare outputs.

## Summary

- Ring‐tailed lemurs fed chopped food spent more time foraging than when fed whole food.
- More locomotion was present when lemurs were provided with chopped food.
- Food presentation style had no significant effect on overall positive behavioural diversity.
- We recommend a feeding style that utilises both chopped and whole food as the best way of promoting the performance of important natural behaviors.

## Graphical abstract

Zoo‐housed ring‐tailed lemurs benefitted from the provision of whole and chopped food items. With different biologically relevant activities being promoted by both chopped and whole food respectively. Providing chopped food increased time on movement (exploration around the enclosure and foraging) and this may have wider health and welfare benefits.

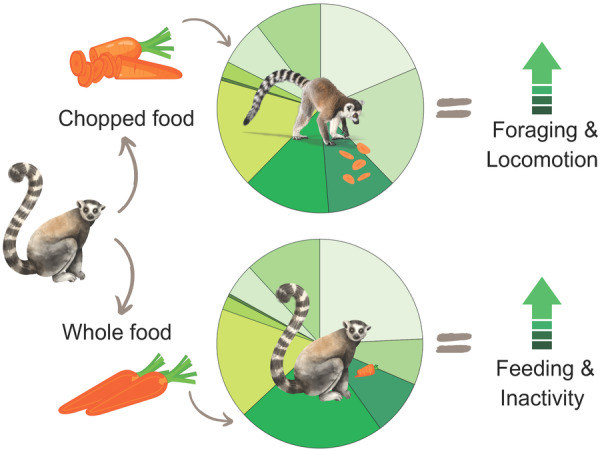

## 1 Introduction

Behavioral diversity can refer to the number of different behaviors performed by an individual animal and the proportion of time spent on one behavior compared to others (Miller et al. 2020). When interpreted correctly this can be a useful measure of naturalism or normality of time activity budgets for zoo‐housed species (Miller et al. 2020). Many species spend a large proportion of their day feeding and foraging. Therefore, it is important to understand the impacts of dietary provision and food presentation on behavioral outputs and how captivity and management routines influence activity patterns (Rose and Riley 2021). It is also suggested that behavioral diversity is enhanced if animals experience positive events in their captive environments (Hall et al. 2021). Therefore, measuring behavioral diversity in relation to husbandry (such as feeding procedures) can help assess the welfare state of animals. Results from such observations can then determine if changes need to be applied to encourage natural behavior and prevent issues, such as undesirable aggression or abnormal behaviors, occurring or developing further.

Scattering chopped food in a zoo animal's enclosure is a common feeding method and a form of environmental enrichment (Young 1997). The practice has been used extensively to promote positive welfare outputs across species as scatter feeding increases time spent on exploration and foraging (Bennett et al. 2014; Dishman et al. 2009). Working for a resource even when such a resource is easily available, termed “contrafreeloading” (McGowan et al. 2010), can promote a wider suite of foraging behaviors that are beneficial attaining positive welfare outcomes (Troxell‐Smith et al. 2017; Waasdorp et al. 2021). However, the practice of chopping food for a range of captive animals has been questioned (Shora et al. 2018; Griffin and Brereton 2021; James et al. 2021) because food presentation differs from how wild food items would be experienced and food preparation could be time consuming. It has also been suggested that providing whole food can allow for increased manual manipulation of food items (Kerridge 2005) and increase feeding time (Smith et al. 1989). However, given that captive primates show enhanced positive behavioral diversity and enhanced levels of foraging and exploration when fed chopped food compared to when provided with whole food or clumped food resources (Waasdorp et al. 2021), best practice approaches may be linked to providing chopped and scattered food. Therefore, it is of interest to assess if chopping food is an appropriate method of food preparation that can promote time spent on foraging, exploration and other activities associated with higher behavioral diversity.

Published work on feeding whole or chopped food appears contradictory, with some research claiming whole food to be preferable despite comparing different sized chopped chunks rather than actual whole food against chopped food (Welsh et al. 2022). Nonsignificant findings (Griffin and Brereton 2021; James et al. 2021) also calls for further research, perhaps cross‐institutional study and the measurement of individual animal responses to diet presentation, before the wider behavioral and welfare benefits of providing whole versus chopped food can be fully realized. Waasdorp et al. (2021) clearly demonstrate the value of a sound experimental design when attempting to determine the behavioral impacts of food presentation. Given burgeoning interest for writing best practice husbandry guidelines across zoo‐housed species and a corresponding need to ensure that valid husbandry evidence is available to support such guidelines, it is important to test and evaluate zoo animal responses to their husbandry and management objectively and thoroughly before wide‐reaching recommendations are made on what is deemed most appropriate care for a species.

The ring‐tailed lemur (*Lemur catta*) is a suitable candidate for investigating the effect of food presentation on captive animal behavior, because it is commonly held in many zoos (Law et al. 2021) and is provided with chopped food in captivity (Dishman et al. 2009; Hansell et al. 2020). In the wild this lemur species typically forages for large food items, such as tamarind tree (*Tamarindus indica*) pods, which exhibit morphological variation in shape (straight or curved) and size (e.g., up to 16 cm long) (Van den Bilcke et al. 2014), so it is questionable whether providing chopped food is the correct approach for enabling wild‐type food handling skills in a zoo setting.

Research has suggested that larger food items may be a more suitable option for feeding lemurs (Welsh et al. 2022), but to what extent different feeding methods promote both behavioral diversity and foraging time in this species has yet to be determined. Such information will enable institutions to select the most suitable feeding method for a species to promote good animal welfare and maximum efficiency of the zoo's workforce.

The aim of this study was to analyze activity budgets and behavioral diversity when ring‐tailed lemurs were presented with either chopped or whole food and to assess if style of food preparation is an important factor that influences time spent on natural foraging. Such data would help develop recommendations that could promote performance of natural behaviors and increase behavioral diversity displayed by captive ring‐tailed lemurs. Although lemurs forage for whole items in the wild, we predicted that whole food items would result in reduced time spent on foraging behavior and behavioral diversity when compared to chopped food items because they are less likely to take time to search for food. As wild food items may be provided in a glut (e.g., on a fruiting tree) and thus be readily available to all troop members, a smaller number of larger food items in a zoo enclosure may not replicate the choice and opportunities for individual foraging, thus resulting in reduced foraging time.

## 2 Methodology

### 2.1 Study Site and Subjects

We conducted observations of ring‐tailed lemur behavior at Tropiquaria Zoo, Somerset, United Kingdom over a 12‐week period from November 1, 2022 to January 25, 2023. The lemur enclosure consists of inside and outside on‐show and off‐show areas, and the lemurs always have access to the outdoor areas unless weather is severe. Artificial climbing structures (fire hose), wooden feeding platforms, and burlap hammocks are included in the indoor areas. Hay is used as a bedding substrate for the hammocks, and wood shavings are used as floor substrate. Indoor houses are heated to 18°C–26°C, dependent on seasonal conditions. Artificial climbing structures (wooden posts and fire hoses), swinging wooden platforms, and natural branches, logs and tree stumps are included in the outdoor areas. Both outdoor areas have a grass substrate, along with sparse gorse shrubbery. A natural conifer thicket covers the back of both outdoor enclosure areas, which provides shelter and climbing opportunities. The enclosure is not a walk‐through exhibit, and a safety barrier prevents visitor contact with lemurs.

During the study period, we observed the entire population of six, unrelated, captive‐bred ring‐tailed lemurs within the enclosure. The individuals were between three and 18 years old and the population consists of a sexually mature female, four sexually mature males, and a geriatric male (Table 1).

**Table 1:** Ring‐tailed lemur subjects observed in the study.

Lemur Subject	Sex	Age (years)	Reproductive Status
A	Male	18	Geriatric male (not part of breeding program)
B	Male	4	Sexually mature male (not part of breeding program)
C	Male	3	Sexually mature male (not part of breeding program)
D	Male	14	Sexually mature male (not part of breeding program)
E	Male	14	Breeding male (part of breeding program)
F	Female	16	Breeding female (part of breeding program)

### 2.2 Food Preparation

We maintained normal feeding times and husbandry routines throughout the study. During the study, we presented food in two different delivery methods: (1) food chopped into one‐inch cubes, and (2) whole food items. Chopping food is the normal method of food delivery at the zoo, but to reduce any bias in how lemurs responded to dietary presentation and to maintain appropriate dietary composition, the quantity and variety of food allocations remained the same regardless of food presentation method. For both conditions produce consisted of 72 g of root vegetables (40% of diet), 72 g of leafy vegetables (40%), 18 g of non‐citrus fruit (10%), and 18 g of foraged greens (10%). Both whole food items and chopped food were scattered randomly across the animals' enclosure—food was not provided in specific discrete clumps or measured distributions within the enclosure, nor in enrichment items that would also extend feeding time.

### 2.3 Data Collection

Before observations, we defined lemur behavior in an ethogram that was used as a basis for recording behaviors during the observations (Table 2). The behaviors were selected based on observations of the lemurs and background research conducted on the behavioral repertoire of strepsirrhine primates (Clutton‐Brock 2012; Collins et al. 2017).

**Table 2:** Ethogram of ring‐tailed lemur behaviors used for observations. Behaviors used in final analyses are presented in italics.

Behavior	Description
*Feeding*	Consumption of food items.
*Foraging*	Searching for food items in the enclosure.
*Locomotion*	Terrestrial and/or arboreal movement in and around the enclosure using hands, feet, and tail.
*Social*	Directed interactions from one individual lemur to another, indicative of communication or sociality. Including huddling behavior (where a group of lemurs all collect together, with bodies touching).
*Maintenance*	Self‐grooming using tongue and paws, and sun‐bathing (body orientated towards direct sunlight).
Aggression	Species‐typical aggressive behavior including dominance and submissive displays, conflict and direct physical contact.
*Inactive*	Sleeping or resting in a relaxed state with minimal to no distinctive movement with eyes open or closed.
Abnormal behavior	Repetitive action that is not part of the natural behavioral repertoire, including pacing, head tossing, and/or self‐clasping.
Hiding/out of sight	Hidden out of view behind a physical or visual obstruction, either intentionally or unintentionally.
Alert	Distinct observational behavior with hackles raised and/or intense staring in a particular direction with a restless demeanor.
Human‐orientated behavior (visitor/keeper)	Any form of behavior orientated to keepers or visitors rather than conspecifics.

We carried out instantaneous focal sampling of each lemur for a period of 30 min starting after a 5‐min period for the animals to habituate to the presence of the observer, two times daily (08:00 and 14:00). These timings were chosen due to them coinciding with the typical feeding times, and data collection occurred as soon as the group was fed (allowing for the 5‐min habituation period). We carried out twelve observations per individual (three morning and three afternoon observations under each condition) and we recorded behaviors at 1‐min intervals. Before observations, we recorded temperature and weather conditions, and during observations we noted visitor presence (counting the number of visitors present at the enclosure), because these were factors that could potentially affect foraging behavior. We considered these co‐variates to help determine if food‐type was the main factor impacting foraging.

### 2.4 Statistical Analysis

We calculated Shannon Diversity (*H*‐index) on behavioral observations in both food preparation conditions using Past v.4.11 statistical software (Hammer et al. 2001). This diversity index has traditionally been used for assessing ecological communities (Morris et al. 2014), but it has been adapted here behavioral diversity whereby a higher value of *H* represents higher levels of diversity (Chao and Shen 2003). The *H*‐index uses the number of behaviors seen and the proportion of time spent on each, compared to the overall observation time. The equation for the H‐index used for this study is the proposed revision for behavioral data in the statistical package “Past” (Hammer et al. 2001).

$$H=-\sum \left(p_{t}*\;\ln\left(p_{t}\right)\right)$$

Where *H* represents the Shannon Diversity Index. p_t_ is the proportion of time spent on each behavior divided by the total proportion of time spent on all behaviors. ln(p_t_) is the natural logarithm of p_t_. −Σ is where the result of the p_t_ * ln(p_t_) calculation for each behavior is summed and then multiplied by −1.

The unbiased version was selected on Past to reduce bias caused by small samples (Hammer et al. 2001). This uses the following formula which we have adapted for behavioral data:

$$H_{u}=H+\left(B-1\right)/\left(\text{2n}\right)$$

Where *H*_u_ is the unbiased *H* value. B‐1 is the number of different behaviors minus 1. 2n is the total proportion of time spent on behaviors multiplied by 2.

If diversity is high, the animal is performing a range of different behaviors for an even amount of time across all those observed. If diversity is low, then the animal is performing a lower number of behaviors and is spending more time on one or two behaviors and not on all noted. Some behaviors may have been performed momentarily if at all. If one behavior dominants during the observation period, there is low overall behavioral diversity (Miller et al. 2020).

A limitation with assessing behavioral diversity is that it does not always consider whether behaviors reflect positive or negative welfare (Cronin and Ross 2019; Tallo‐Parra et al. 2023). Therefore, we selected behaviors that we deemed to be positive from the observations for these analyses (feeding, foraging, locomotion, social, maintenance, and inactive as a measure of comfort) and all other behaviors in the ethogram were omitted, as per Miller et al. (2020). A Wilcoxon Signed Rank Test was used to determine if there was greater behavioral diversity when lemurs were presented with chopped food compared to whole food and a Kruskal–Wallis test was used to test whether there was a significant difference in the number of positive behaviors between delivery methods. All analyses were conducted in Minitab version 21.4.2 (Minitab LLC 2021).

Based on the outputs from descriptive analyses, lemur time activity budgets (for feeding, foraging, locomotion and maintenance behaviors) were tested for any significant effect of food condition using a repeated measures ANOVA in Minitab. We included lemur ID as the random factor and food presentation style (chopped/whole) as the fixed factor.

To determine which factors influenced foraging behavior, we further ran a repeated measures ANOVA in Minitab with food presentation type (chopped or whole), weather (cloudy, sunny, rain), temperature (°C), and visitor presence as fixed factors and lemur subject (A–E) as a random factor. The response variable was transformed by arcsine transformation because data were proportions, and an Anderson Darling normality test was carried out in Minitab to determine if the repeated measure ANOVA method was suitable. We also calculated the partial r^2^ for each significant predictor from this ANOVA to define how much variation is accounted for by each significant predictor.

The raw data set from this study is available here https://figshare.com/s/b9f093751d809e5d8342.

## 3 Results

### 3.1 Activity Budget

**Figure 1:**
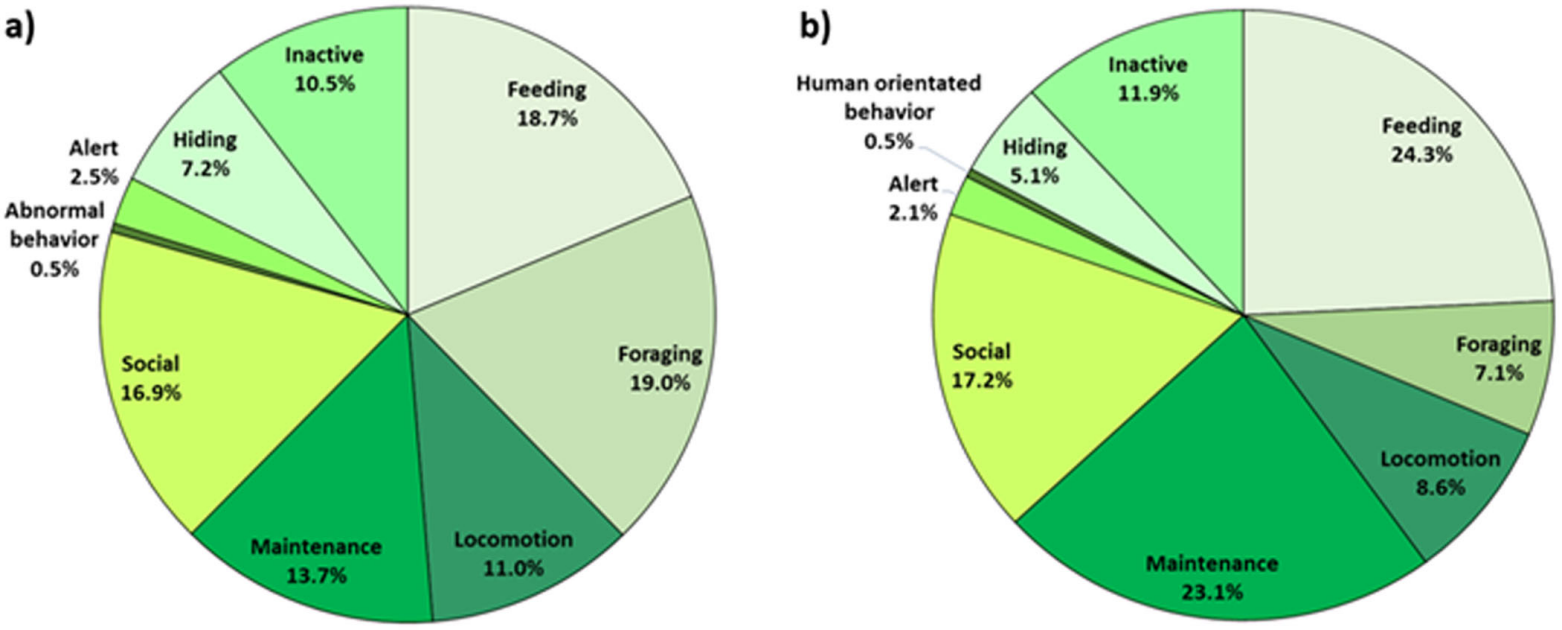
Activity budget of ring‐tailed lemur population in (a) chopped food condition; (b) whole food condition. Percentages are derived from total observations in each condition for all animals combined.

**Figure 2:**
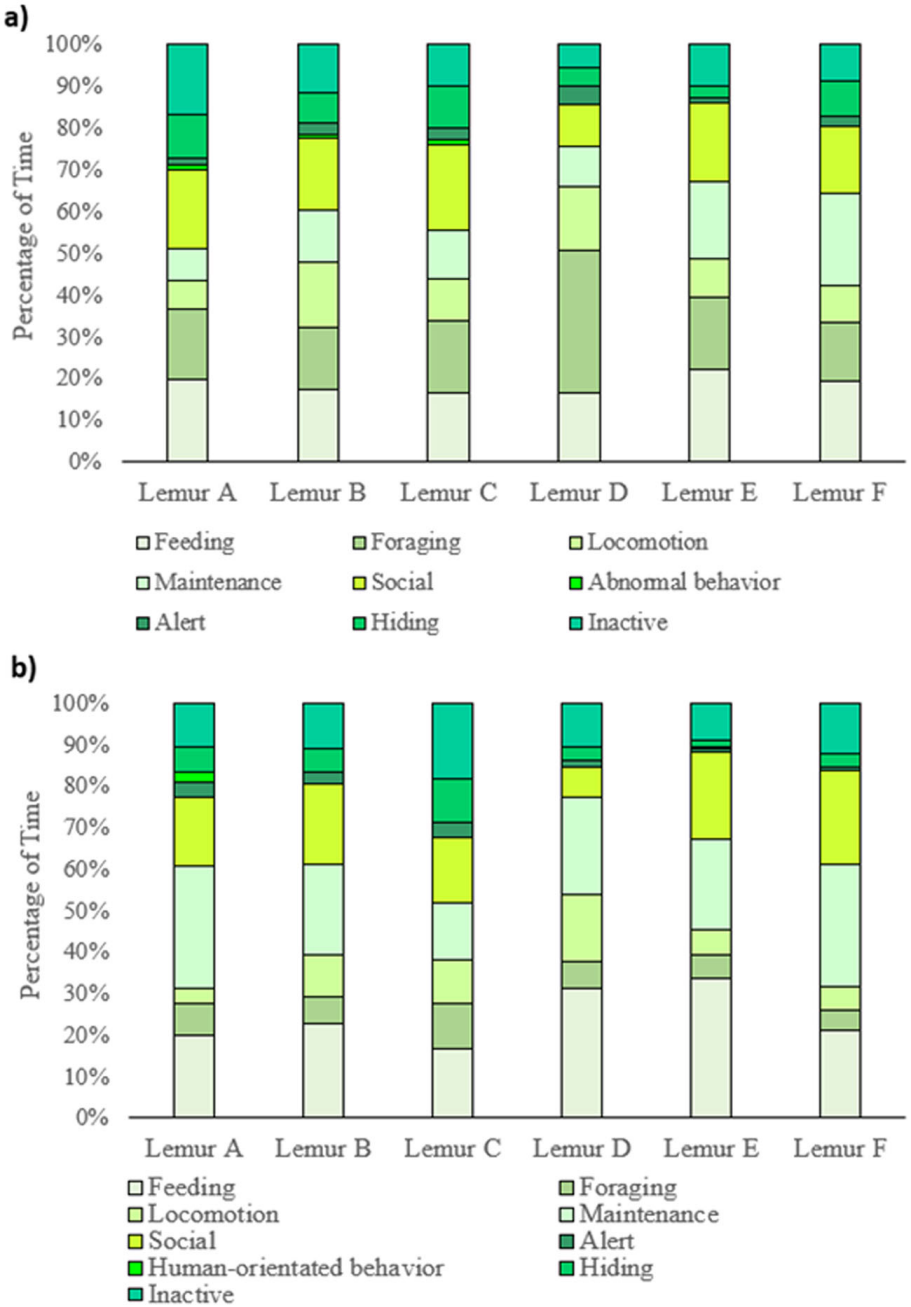
Activity budget of individual ring‐tailed lemurs in (a) chopped food condition; (b) whole food condition.

More foraging (*F*_1, 65.68_ = 44.16; *r* ^2^ = 0.46; *p* < 0.001) was observed with chopped food (19.0% of the time) than with whole food (7.1%). Figure 1 shows that the commonest behavior of the population during the whole food condition was feeding (*F*_1, 65.25_ = 5.6; *r* ^2^ = 0.16; *p* = 0.021) – 24.3% compared to 18.7% in the chopped food condition. Aggression did not occur in either condition. Other behaviors, including social activity, were similar across conditions, and locomotion was higher in the chopped food condition (11.0% compared to 8.6% in the whole food condition), but this was not significant. Maintenance behaviors were higher in the whole food condition (*F*_1, 65.38_ = 9.94; *r* ^2^ = 0.14; *p* = 0.002) – 23.1% compared to 13.7% for chopped food. Rates of abnormal and human‐orientated behaviors were zero to negligible (occurring for less than 0.5% of the time) across all food presentation conditions. Individual lemur activity budgets are presented in Figure 2.

### 3.2 Behavioral Diversity

**Figure 3:**
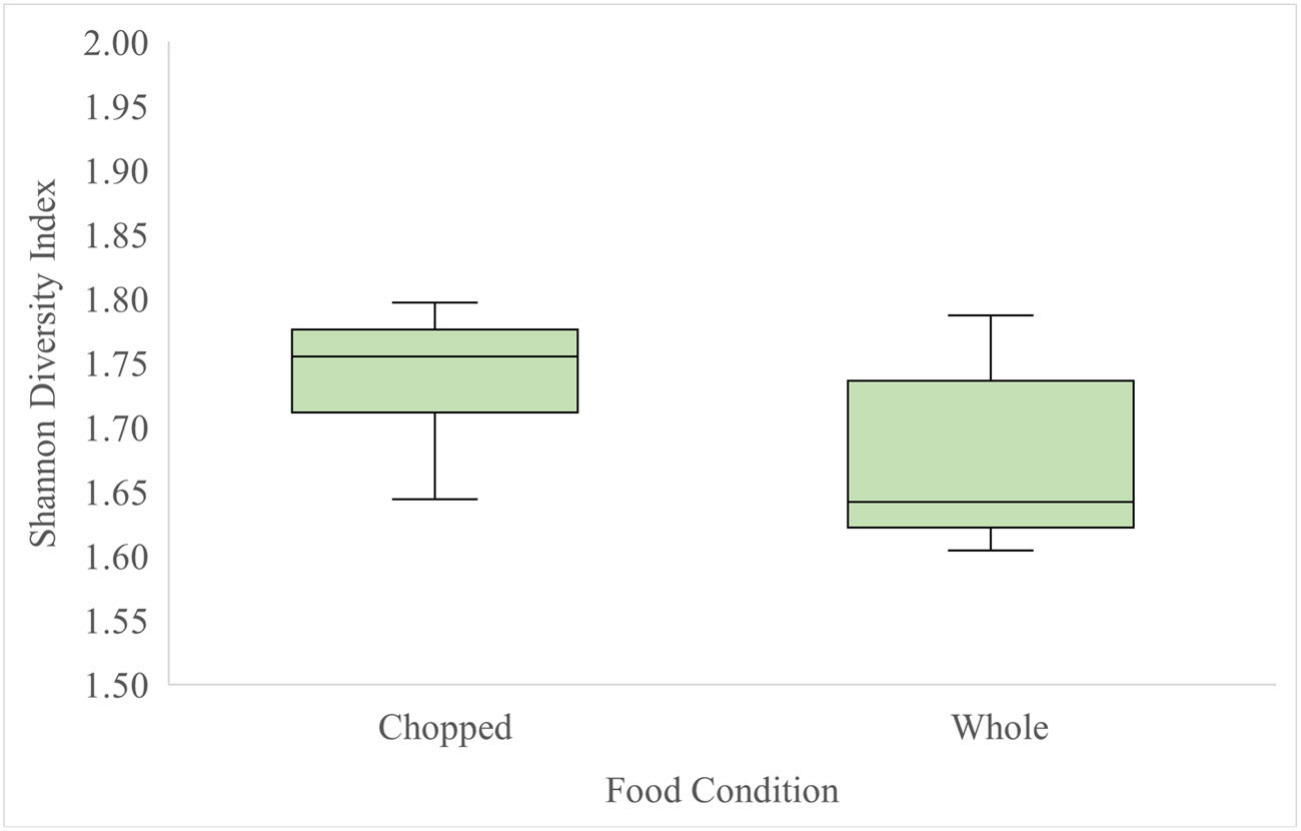
Median Shannon Diversity for chopped and whole food conditions for lemurs. Boxes show interquartile range and whiskers represent high and low values.

**Figure 4:**
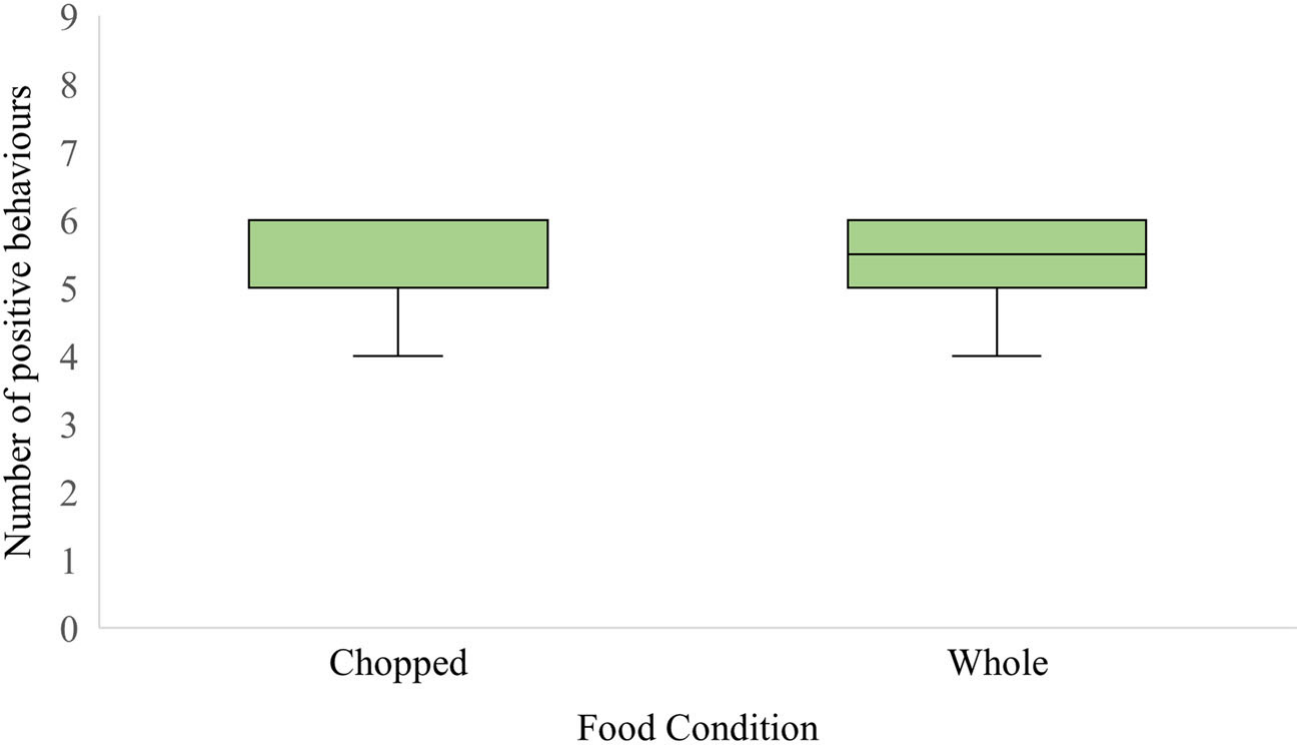
Median number of positive behaviors (feeding, foraging, locomotion, social, maintenance, and inactive) for chopped and whole food conditions for lemurs. Boxes show interquartile range and whiskers represent high and low values.

In the chopped food condition, the median Shannon Diversity Index value of all individuals was 1.755. In the whole food condition median Shannon Diversity Index value of all individuals was 1.642 (Figure 3). The lemurs did not exhibit significantly greater behavioral diversity (in terms of time spent across behaviors and the number of behaviors observed) when presented with chopped food compared to whole food (Wilcoxon Signed Rank Test: *W* = 3.00, *p* = 0.142), and there was not a significant difference between the number of different positive behaviors observed in each food condition (Kruskal–Wallis Test: *H* = 0.11(1), *p* = 0.739 adjusted for ties) (Figure 4).

### 3.3 Factors Influencing Foraging Behavior

**Figure 5:**
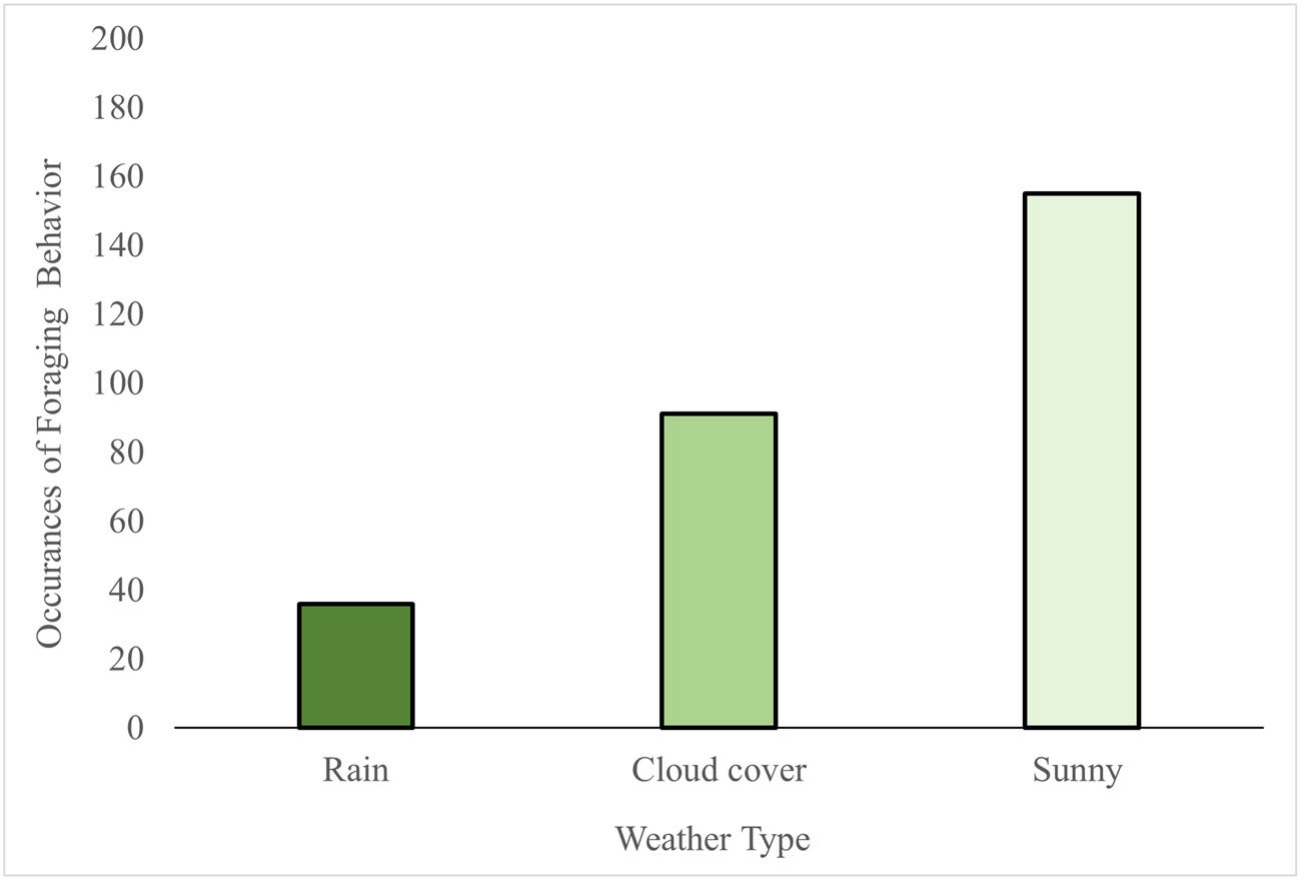
Total occurrences of foraging in rain, cloud cover and sun during behavioral observations in both chopped food and whole food conditions.

Table 3 shows that food presentation type had a highly significant effect on foraging behavior (*p* < 0.001) in this study, and weather was also significant (*p* < 0.042). There were more occurrences of foraging behavior with chopped food, and when weather was sunny compared to cloud cover or rain in both chopped food and whole food conditions (Figure 5). Temperature, lemur subject, and visitor presence did not have any effect on time spent on foraging behavior throughout the study period. The *r* ^2^ value for this repeated measures ANOVA was 0.37 suggesting that a further 0.63 of variation impacting lemur behavior is not captured by this test. The partial *r* ^2^ for food presentation style (not including weather) was 0.31, therefore weather and other factors are accounting for 0.69 of the unaccounted variation.

**Table 3:** Repeated measures ANOVA results for factors in relation to foraging behavior. Significant effects are asterisked.

Factor	*F*‐value	DF (Error)	*p*‐value
Food presentation type	23.72	1 (48)	< 0.001*
Weather conditions	3.40	2 (48)	0.042*
Temperature	0.43	8 (48)	0.895
Visitor presence	0.19	1 (48)	0.662
Lemur subject	1.05	11 (48)	0.417

## 4 Discussion

The results from this study show that providing chopped food to a group of zoo‐housed ring‐tailed lemurs increased time spent foraging and time spent moving around the enclosure. In this regard, our research supports that of Waasdorp et al. (2021) by showing the positive welfare benefits accrued by captive primates when fed chopped food. When provided with whole food items, lemurs spent more time feeding and performed more maintenance behavior. Rates of abnormal and human‐centered behaviors were very low in overall performance across conditions.

### 4.1 Behavioral Diversity

There was no significant influence of food presentation style on positive behavioral diversity (more time spent on more behaviors for one food type compared to the other) even though we calculated higher positive behavioral diversity for chopped food, thus further data collection is needed to validate any causal effect. Chopped food items may provide more opportunity for lemurs to display a wider diversity of behaviors for longer periods, but as the difference between food presentation conditions was not significant, and there was not a difference in the number of different positive behaviors observed between the two delivery methods we cannot show any causal link between chopped or whole foods and enhanced positive behavioral diversity.

Compared to baseline (chopped food), the calculated H‐index for whole food was almost as high; this is important because a diverse behavioral repertoire in captivity is linked to experiences of improved welfare (Miller et al. 2020). High behavioral diversity may help reduce the chances of abnormal behavior performance as increased positive behavioral diversity can indicate environmental suitability (i.e., housing in an environment that promotes the performance of many different behaviors). Regardless of food presentation style, the enclosure for these lemurs is likely to have been suitable for their needs and requirements. It is likely that other aspects of the animals' enclosure design, social environment, and husbandry routine (e.g., the presence of environmental enrichment) are more important and impactful influences on how these lemurs dedicate time to different behaviors, thus reducing the overall effect of diet presentation style.

### 4.2 Activity Budget

Foraging was most observed for chopped food (19.0%), which may be because smaller pieces of food are harder to locate, requiring a longer duration of search time (Young 1997). This is similar to findings by Waasdorp et al. (2021) where it was found that dispersing chopped food for captive white‐naped mangabeys (*Cerocebus lunulatus*) increased foraging behavior. Smaller food items are more easily distributed across the enclosure, resulting in a greater range of locomotory, exploratory and foraging behaviors (Yamashita 2008). During the whole food condition, foraging only accounted for 7.1% of the activity budget, which is more likely to occur because whole food items are easy to locate. In the long‐term this may reduce activity, leading to obesity and subsequent health issues, thus reducing the physiological well‐being of the animal (Goodchild and Schwitzer 2008).

Under both feeding conditions, performance of abnormal repetitive behavior was negligible to zero. This may be a characteristic of this specific group of lemurs and therefore further research should consider multi‐population study to quantify any meaningful impact of food presentation type on reduction of abnormal behavior and promotion of species‐typical activities. Whole food items also increased time spent feeding (i.e., consuming food) and on maintenance behaviors, potentially reducing the animal's wider engagement with the environment. However, these very low rates of abnormal behavior that occurred around feeding time means it is unlikely that any underlying welfare concerns related to husbandry and animal management are apparent.

Aggression was not observed in either food presentation condition. The lack of this response in both conditions may be due to the amount of food fed and the area that food was distributed over, with all lemurs being able to find an area of the enclosure to forage in. The stability of the social group is also likely to have influenced rates of aggression, and we recommend replication of our study in other troops of different sizes and demographics to further interpret the effects of feeding style on social interactions. We might have expected aggression to occur when lemurs were provided with whole food due to increased competition for more desirable items, and that larger pieces of food may have more intrinsic value than smaller items (Mathy and Isbell 2002). However, other research has found that aggression can be lower when whole food is presented to primates (Sandri et al. 2017) and this may be because animals are more occupied processing the food item they have selected. The nonlinear dominance hierarchy and complex web of social interactions within a ring‐tailed lemur troop (Nakamichi and Koyama 1997; Sauther et al. 1999) may mean that animals in this lemur troop know who to defer to when selecting food items, as well as who they can displace from a valued resource. This hierarchy and inbuilt understanding of associates may explain the limited aggression displayed by these lemurs during each food presentation condition.

### 4.3 Factors Influencing Foraging Behavior

Food presentation type was the most important factor that influenced foraging behavior in this study. This suggests that the method of food preparation has a significant influence on whether the lemurs foraged for longer when presented with different styles of food presentation. Weather also had a significant influence over behavior, and this is unsurprising as previous research shows that lemurs rest more in rainy conditions at the expense of other activities such as foraging (Goodenough et al. 2019). Our data show that foraging was recorded less frequently during rain because the lemurs were sheltering indoors, whereas on sunny days lemurs spent more time foraging outdoors. However, this impact of weather was not as significant as the effect of the food presentation itself on foraging. Temperature did not influence foraging, but as all observations took place over winter, the research should be extended to determine any impact of varied temperatures in different seasons, such as summer, on lemur activity and engagement with food items.

Visitor presence did not have a significant effect on time spent foraging and this is a positive indicator of welfare because it implies that this enclosure design has minimized any negative issues associated with the “visitor effect” (Hosey 2000; Sherwen and Hemsworth 2019). Stress caused by visitor presence could result in a reduction of positive behaviors, such as foraging, and the formation of abnormal behaviors (Quadros et al. 2014). Lemurs may be habituated to visitor presence and therefore the mode of food presentation was a more important causal factor for behavioral performance, rather than whether visitors were present.

Ultimately, the low *r* ^2^ value for our testing (37%) suggests that other variables may be responsible for variation in lemur activity and therefore we would recommend further study into individual animal behavior patterns, as well as measurement of enclosure‐specific and wider environmental variables that have not been considered in this study. Measurement of nutritional factors in the feed of the lemurs, including palatability, energy density and individual animal preferences could also be investigated to elucidate any difference (or lack of) in behavioral diversity related to food presentation and the actual perceived quality of the food by the animal. Understanding what causes satiation in lemurs may be important, as research notes that consumption of multiple smaller meals gives greater control over satiety and therefore chances of moving on to a new or different behavior post‐consumption (Schwitzer and Kaumanns 2003). Therefore, chopped, scattered food may provide more choice and control over the animal's use of resources and subsequent behaviors patterns—as well as providing opportunities for positive challenge (Rose and Lewton 2025)—that supp—ort enhanced welfare outputs.

Increased time on maintenance behavior when fed whole food is also worthy of further investigation. Many captive species will perform redirected grooming or preening behaviors when opportunities for food manipulation or search time for food are reduced (Meehan et al. 2003; Beisner and Isbell 2009). Therefore, providing different feeding opportunities, using a mixture of whole and chopped foods may be beneficial as this would increase feeding time, yet reduce inactivity by promoting search, exploration, and location of food.

Conflicts over food are a natural part of primate sociality, even though such encounters can be risky (Norscia and Palagi 2011). Providing a mixture of chopped and whole foods could promote a wider range of social interactions and such an intervention is worthy of further research. Encouraging some competition over food may be beneficial for group stability, development of behavioral flexibility and cognitive development via problem solving. Providing different amounts of resources in different areas of the enclosures, and in differing formats, could have wider social benefits that support the performance of wild‐type activity budgets.

Ring‐tailed lemurs are flexible in how and when they forage, depending on seasonality and food availability (LaFleur and Gould 2009). Zoos could use such behavioral ecology information to spread chopped and whole food items around the animal's enclosure at different times of the day to promote variability in resource access, reduce predictability and encourage further opportunities for exploration. Due to the high rates of obesity in captive ring‐tailed lemurs (Mellor et al. 2020), encouraging exercise and slowing down food consumption may be beneficial for longer‐term health. Nocturnal movements (including foraging) may occur on moonlit nights (Parga 2011) and therefore zoos should consider effects of artificial lighting on lemur feeding and foraging behavior, providing opportunities to forage nocturnally on low‐calorific food items a few times per month. Such variation in feeding practice may increase positive behavioral diversity when a lemur's space use may be restricted. Given there were times during our observation schedule that animals were choosing to remain indoors, enhancing such inside spaces to make them more suitable to performing a wider range of activities could improve lemur welfare.

A main cause of obesity in captive ring‐tailed lemurs is overconsumption (Pontzer 2023). Given that animals in our study moved less and ate more when provided with whole food, scattered food over a wider area may promote longer‐term animal health and better body condition. We recommend further research that observes lemur responses to diets with different proportions of whole and chopped food items to determine which form of food presentation promotes the widest behavioral diversity, longer time spent foraging, exploring, and moving around the enclosure, and enables all animals access to resources without prolonged competition or experiences of aggression. Extension to this study on activity levels and food presentation could also focus on how food is presented—that is, randomly dropped compared to presented in discrete clumps. In this way, chopped food presentation could mimic a larger piece of whole food and resulting impacts on behavioral diversity and time‐activity budgets be measured. Dishman et al. (2009) shows how zoo‐housed lemur activity is influenced by how different types of food are presented (in terms of spatial distribution and variety)—therefore, manipulation of what foods are presented, and how, as well as measuring individual preferences could provide useful evidence for how to vary feeding regimes to enhance species‐typical behavioral diversity.

To further improve primate health and wellbeing, zoos should focus their attention on frequent assessment of dietary nutrients, species metabolic needs, energetic content and animal intake to ensure species‐appropriate nutritional requirements are met (Donadeo et al. 2016). Maintenance of appropriate social groups, as this impacts on time spent foraging and access to resources (Junge et al. 2009; Teague O'Mara 2015) and providing a complex environment that provides for a species' behavioral needs, can also promote naturalistic foraging and exploration times (Junge et al. 2009; Laméris et al. 2021). Utilizing a mixture of food presentation types of prepared and whole produce, may be the best way to achieve this. Research should then focus on how this variety in food presentation enhances time‐activity patterns and promotes positive welfare states rather than attempting to determine the mutually exclusive benefits of chopped vs. whole dietary items. It is essential that research reporting on the effects of whole food diets is clear and unambiguous in the description of food preparation and how food was actually provided to the study animals to ensure valid and objective consideration of resulting behavioral outputs. We suggest developing our methods, to calculate individual lemur behavioral diversity at different times of the day, across different seasons and with different degrees of human presence. A limitation of our study, and one that has meant we have failed to identify significant change in behavior with food presentation type, could be the lack of specificity to our ethogram. Therefore, ethogram refinement that deconstructs each state behaviors into smaller constitute parts—as demonstrated in other behavioral diversity research (Miller et al. 2020)—could help more precisely identify change in a lemur's behavioral when different food presentation is offered. Changing our choice of index, to the 1‐Simpson's Diversity Index, for example, could also provide more robust diversity scores, which do change with food presentation condition, as this index copes better when some behaviors are performed more commonly than others (Hall et al. 2021).

## 5 Conclusions

Our research shows that chopped food being scatter fed is an appropriate feeding method for captive ring‐tailed lemurs because it promoted more time on foraging activity. However, whole feeding should also be utilized because it provides variation in dietary presentation, which can potentially reduce the likelihood of abnormal behavior performance and provide stimulation for a wider range of other food‐orientated behaviors. Our findings on effects of whole or chopped food provision on lemur positive behavioral diversity showed no significant difference between conditions and therefore other factors are likely more impactful influences on how lemurs partition their time to different behaviors. Our study recommends that both methods should be used together to provide more opportunities for exploration and locomotion by incorporating scatter feeding of chopped food, and to allow lemurs to spend time eating by occasionally offering them whole food.

## Ethics Statement

This study was approved by an ethics committee in the Animal & Environmental Sciences department of Bridgwater and Taunton College on 21st October 2022, and all methods were agreed by Tropiquaria Zoo in advance of data collection.

## Conflicts of Interest

The authors declare no conflicts of interest.

## Data Availability

Data has been uploaded into the repository Figshare and will be made public at acceptance.
